# Parent and Teacher Depictions of Gender Gaps in Secondary Student Appraisals of Their Academic Competences

**DOI:** 10.3389/fpsyg.2020.573752

**Published:** 2020-09-25

**Authors:** Milagros Sáinz, Sergi Fàbregues, Jordi Solé

**Affiliations:** ^1^Internet Interdisciplinary Institute, Universitat Oberta de Catalunya, Barcelona, Spain; ^2^Faculty of Psychology and Education, Universitat Oberta de Catalunya, Barcelona,Spain

**Keywords:** gender, self-perception of ability, socialization, stereotypes, STEM

## Abstract

The present study examines a group of secondary teachers’ and parents’ appraisals of gender gaps in secondary students’ self-assessment of competence in Science, Technology, Engineering, and Mathematics (STEM) and non-STEM domains. Eight focus groups with 39 parents and 34 secondary teachers were conducted for this purpose. In light of the observed gender gaps in students’ performance and self-perception of ability in the different subject areas, the participants were particularly surprised by girls’ underestimation of their abilities in STEM subjects, compared with boys’ tendency to overestimate their abilities in STEM. Most participants agreed on the need for measures to combat these inaccuracies and discussed possible causes. Some participants associated these gender disparities in students’ self-assessment of ability with gender gaps in their choices of subject areas and occupations. The role played by school, teachers, families, and other socialization agents in reinforcing gender stereotypes about academic competence was also discussed in most of the focus groups. Interestingly, some teachers questioned why gender attainments obtained in schools do not serve as an example when it comes to neutralizing the sexism and gender inequality messages offered by the media and society. Likewise, technology teachers proposed changes in school practices to close gender gaps in certain areas (i.e., boys’ appropriation of the playground, or the reproduction of gender roles in the classroom). Few parents acknowledged their unconscious reproduction of gender roles and stereotypes in raising their children.

## Introduction

A recent meta-analysis of studies carried out between 1914 and 2011 in more than 30 countries (70% of the sample comprised students from the United States) concluded that girls have systematically achieved higher academic performance than boys for nearly a century (Voyer and Voyer, 2014). This study also concluded that, though boys tend to score higher in math and science on standardized tests such as the OECD’s PISA test, girls achieve higher school grades in all subject areas (Voyer and Voyer, 2014). Interestingly, the female advantage in school performance in math and science seems not to become apparent until junior or middle school (Voyer and Voyer, 2014). According to this meta-analysis, the widest gender differences were reported for language courses and the narrowest gap was recorded for math and science. These differences increased from elementary to middle school but declined across post-secondary compulsory school (Voyer and Voyer, 2014).

The generalized nature of the female advantage in school performance contradicts the existing stereotypes that girls excel exclusively in languages, while boys only excel in math and science (Tiedemann, 2000; Skaalvik and Skaalvik, 2004; Sáinz and Eccles, 2012). According to a recent study, girls’ better use of extra time at the end to finish tests on account of their ability to sustain performance can explain gender differences in score performance (Balart and Oosterveen, 2019). Unfortunately, according to Voyer and Voyer (2014), women’s better performance over that of their male counterparts throughout compulsory schooling in most countries has received little attention as a global phenomenon.

To the authors’ knowledge, there is currently a lack of research into parents’ and teachers’ views of these gender differences. In order to fill this gap in the literature, this qualitative study aims to analyze the opinions of parents and teachers on this issue.

### Gender Disparities in Students’ Appraisal of Their Academic Abilities

Young people’s self-perception of ability plays a major role—even higher than actual performance—in shaping boys’ and girls’ choices (Sáinz and Eccles, 2012; Bian et al., 2017). According to the expectancy-value theory of achievement motivation, students will choose courses of study for which they have an expectancy of success and which have a high value for them (Eccles, 2007). During recent decades, much research has been devoted to evaluating students’ discrepancies in self-assessment of their academic competences in different subject areas (Guimond and Roussel, 2001; Gonida and Leondari, 2011; Sheldrake, 2016). Some of these studies have examined gender disparities in the way girls evaluate their abilities in Science, Technology, Engineering, and Mathematics (STEM) subjects such as math, technology, and physical science (Sáinz and Upadyaya, 2016; Sheldrake, 2016). In this regard, girls tend to systematically undervalue their academic competences in STEM domains such as math, physical science, and technology, despite the fact that their scores are equal or higher than those of their male counterparts (Sáinz and Eccles, 2012; Sáinz and Upadyaya, 2016; European Parliament, 2020). Strikingly, boys tend to consistently overestimate their competences in these STEM subjects (Guimond and Roussel, 2001; Sáinz and Eccles, 2012).

The field of STEM has been the focus of an immense body of research into gender gaps in self-perception of ability. There is a widely held belief that STEM degrees and careers are difficult and that a student needs to be brilliant to enter and succeed in these fields (Shin et al., 2016). These gender differences in perception of intellectual ability emerge at an early age (Bian et al., 2017). Six-year-old girls were more likely to attribute brilliance to males and less likely to report interest in the game labeled for brilliant children than in the one labeled for hardworking children. If girls do not see themselves as clever enough for STEM, they will not develop interest in STEM subjects. This could explain why girls do not enroll in studies and occupations requiring high mathematical abilities, despite having the potential to do so. If women are to engage and take an active role in STEM disciplines, then it is crucial to change women’s and girls’ self-perception of ability (Wang et al., 2017) and self-efficacy (Adams et al., 2014) in relation to STEM, especially in areas where women have traditionally been underrepresented such as technology, physical science, and computer science.

Students’ accuracy in the evaluation of their academic competences predicts different motivational indicators, such as students’ interest in science and STEM (Sáinz and Upadyaya, 2016; Sheldrake, 2016), performance in various subjects (Bouffard et al., 2011), or achievement goal orientations (Gonida and Leondari, 2011). However, to the authors’ knowledge, there is a lack of research into the ways in which parents and secondary school teachers interpret these gender gaps in students’ evaluation of ability in different domains, particularly in STEM subjects. Gaining further understanding about these issues may help promote more realistic assessments of students’ abilities and prevent girls and boys from having negative and positive respective beliefs about their potential in STEM subjects.

### Gender Socialization Influences of Parents and Teachers on Students’ Appraisal of Scholastic Competences

Individual differences in competence beliefs, cognitive capacity, and interests are shaped by students’ experiences in broader sociocultural contexts, at home and school, for example (Eccles, 2009). Gender-role socialization can lead females to have less confidence in their abilities than males (Eccles, 1987, 2007). For instance, parents shape their children’s perception of their ability, thus influencing the choices they make, by providing dissimilar messages about their ability in different domains (Frome and Eccles, 1998; Eccles, 2007).

Research into the socialization of expectations like the expectancy-value theory of achievement motivation has tended to focus on attributional processes and on the differential treatment received by boys and girls, at home and in the classroom (Eccles, 1987, 2007). According to this research, many parents and teachers draw on gender stereotypes regarding boys’ and girls’ abilities, and they communicate these beliefs through various indirect and explicit behaviors (Frome and Eccles, 1998; Eccles, 2007). Girls are therefore considered to be better at English than boys, whereas boys are considered to be better at math and other STEM domains (Eccles, 1987; Sáinz and Eccles, 2012; Sáinz and Upadyaya, 2016). In addition, girls are thought to work harder to master math and other STEM subjects than boys, and vice versa for languages. These gender-differentiated beliefs persist even when school performance levels are controlled (Eccles, 2007). Moreover, students’ self-concepts in different subject areas may also be affected by the extent to which they receive recognition of their achievements or encouragement from teachers, family, and friends to excel at different domains. Girls often tend to receive less recognition and encouragement than boys (Mujtaba and Reiss, 2012).

In addition, teachers as well as students and parents may hold different beliefs about intellectual abilities (Burusic et al., 2011). Some may believe in the so-called fixed mindset, whereby people have different levels of intellectual ability which are basically unchangeable and cannot be modified through effort. Others may believe in the growth mindset, according to which intellectual abilities can be cultivated and developed through effort and instruction (Dweck, 1999). It is therefore likely that parents as well as teachers also differ in their beliefs regarding the origins of individual differences in competence, the meaning of failure, and the most adaptive responses to failure (Eccles, 2007): “These beliefs should influence both their response to children’s failures and their efforts to help boys and girls acquire new competencies and interests” (Eccles, 2007; p. 673).

On the other hand, teachers’ expectations of pupil performance have a major impact on their pupils’ final scores, as observed in the classical experiment conducted by Rosenthal and Jacobson (2003), who coined the term “Pygmalion effect” to observe the influence of self-fulfilling prophecies. Jussim et al. (1996) found that teacher expectations had a significant effect on sixth-grade students’ grades and performance on a standardized mathematics test. Teacher expectations were lower for girls and low-income students. When teachers hold high general expectations for student achievement and students perceive these expectations, students perform better and experience a greater sense of competence as learners (Eccles et al., 1998; Eccles, 1999). In addition, there appears to be a correlation between greater parental encouragement to study math and science and enrollment in advanced math courses, greater interest in math and science, and higher math and science achievement, all of which are predictors of postsecondary education decisions and eventual STEM professional employment (Simpkins et al., 2012; Wang et al., 2017).

### Social, Environmental, and Biological Influences

The impact of stereotype threat is one of the social factors explaining gender differences in school achievement. According to this theory, fear of confirming negative stereotypes about girls’ low competences in math leads girls to perform according to those negative beliefs (Steele, 1997). For instance, teachers who endorse the stereotype that boys are worse at reading or languages than their female counterparts may be less inclined to provide male learners with educational opportunities and resources or motivate male learners to achieve in these areas (Riley, 2014). Similarly, the assumption that girls try harder in school is a positive stereotype that rests on the idea that schools are a better fit for girls than boys. Teachers who hold this positive assumption may behave accordingly and implement educational measures that facilitate girls’ engagement in school.

A number of environmental variables are also associated with symbolic and physical factors that shape the development of gender roles and stereotypes within the school context (Solbes-Canales et al., 2020). From a symbolic perspective, textbooks, teachers, and other educational tools may transmit subtle and implicit messages about what is expected of each student in terms of gender and other sociocultural factors such as parental educational level, social origin, or ethnicity. This is best known as the hidden curriculum (Basow, 2004; Asadullah et al., 2019). Additionally, the physical distribution of the classrooms, spaces, and objects within a school also seems to reproduce the gender differences present in society (Børve and Børve, 2017; Lyttleton-Smith, 2019). Boys, for instance, tend to make greater use of public spaces like playgrounds, engaging in leisure and sporting activities such as football, whereas girls occupy less space in the playground when they play (Karsten, 2003; Clark and Paechter, 2007).

Studies focused on brain differences between boys and girls have shown no statistically significant differences in motivation levels toward science learning between male and female students (Zeyer and Wolf, 2010). However, highly significant differences between personality attributes were observed: female students were more likely to be empathizers (possessing the ability to identify and perceive the mental states of others), and boys were more likely to be systemizers (possessing the ability to understand the world in terms of a system). Students that were more likely to be systemizers possibly had a greater motivation to learn science than empathizers, and the fact that male students were more likely to be systemizers could explain the observed gender differences in male and female students’ choices in relation to STEM.

Given the influence of their beliefs and expectations, together with teachers’ instructional practices, on young people’s achievement and self-perceptions of ability (Eccles, 2007; Wang et al., 2017), understanding the views of teachers and parents is an important step in identifying strategies for reducing gender gaps in school achievement and self-perception of ability. The purpose of the present study is, therefore, to examine parents’ and teachers’ views of gender differences in school performance, self-perceptions, and the poor fit between performance and self-perception of ability. While most of the existing studies addressing these topics have been conducted with self-reported surveys targeting mostly primary and secondary students (Tiedemann, 2000; Bouchey and Harter, 2005), there is a lack of qualitative research providing in-depth analysis of the views of parents and teachers. In order to fill this gap in the literature, we carried out a qualitative study with focus groups addressing the following research questions:

R.Q.1: What are parents and teachers’ views on gender differences in students’ school achievement and self-perception of ability across subject areas?

R.Q.2: What are the differences and similarities between parents’ and teachers’ views on these issues?

R.Q.3: In what ways do the views of teachers in STEM disciplines differ from those of other teachers in non-STEM disciplines?

## Materials and Methods

### Design

A qualitative study was carried out using focus groups. Focus groups were chosen over individual interviews because of their suitability in terms of promoting synergy and enabling meaning-making through dialogue between the study participants. In accordance with their socially oriented nature (Krueger and Casey, 2014), focus groups allowed us to generate interactive contexts of discussion between teachers and parents on the topics of school achievement and adolescents’ self-perception of their abilities. In these interactive contexts, both groups of participants were able to build on their own individual views and experiences and connect them with those of the other participants (Morgan, 1996). Focus groups were productive in eliciting a range of social expectations and norms of teachers and parents, and expressions of agreement and disagreement within and between the groups. These focus groups were conducted between 2016 and 2017 as part of a larger, quantitative, 6-year longitudinal study aimed at examining the development of gendered pathways across secondary school years.

### Participants

Purposive sampling was used to recruit secondary school teachers working at four secondary schools in Madrid and two in Barcelona. Parents of adolescents attending these six schools in the last year of junior high/secondary school were also recruited. We focused on students at this academic level because it is a crucial point in the Spanish educational system, at which students opt for one or another branch of further education. Participants meeting the inclusion criteria were recruited through parent associations and school principals and counselors, who informed them about the study objectives and asked whether they were willing to take part in the study. Following the sample size recommendations in the literature (Guest et al., 2017), a total of eight focus groups were conducted: four of the focus groups were comprised of parents (*n* = 39), and the other four were made up of teachers (*n* = 34). All the focus group samples were homogeneous, that is, composed entirely of either teachers or parents, to ensure the building of shared views. Six focus groups were held in Madrid and two in Barcelona. The characteristics of the participants of each focus group are shown in Table 1.

**TABLE 1:** Characteristics of focus groups participants.

**Demographic**	**FG1**	**FG2**	**FG3**	**FG4**	**FG5**	**FG6**	**FG7**	**FG8**	**Total**
**Gender**									
Male	1	2	3	3	4	1	0	2	16
Female	5	5	7	6	7	9	9	7	55
**Education**									
Primary	0	0	0	0	2	0	0	0	2
Secondary	0	0	0	0	4	3	2	3	12
University	6	7	10	9	5	7	7	6	57
**Type of major**									
STEM	1	3	0	3	3	5	3	3	21
Non-STEM	5	4	10	6	8	5	6	6	50
**Type of participant**									
Parent	–	–	–	–	11	10	9	9	39
Teacher	6	7	10	9	–	–	–	–	32
**Years working as a teacher (only for teachers)**									
Less than 15 years	3	1	1	1	–	–	–	–	6
More than 15 years	3	6	8	8	–	–	–	–	25
**Number of children(only for parents)**									
One child	–	–	–	–	5	2	5	4	16
Two children	–	–	–	–	6	5	4	5	20
Three children	–	–	–	–	0	3	0	0	3
**Gender of children(only for parents)**									
Only male	–	–	–	–	4	3	4	2	13
Only female	–	–	–	–	4	2	5	5	16
Both sexes	–	–	–	–	3	5	0	2	10

### Procedure

The focus groups were facilitated by the principal investigator in quiet classrooms during the evening, after classes were finished, and lasted between 50 and 90 min. Participants received no compensation for their participation. All the focus groups had the same structure. Prior to each focus group session, participants in both groups answered a brief, anonymous, sociodemographic questionnaire eliciting their age and type of studies attained. In addition, while teachers were requested to report the subjects they taught, parents were requested to inform about the number, gender, and age of their children. All the sessions began with a brief introduction of the study aims given by the facilitator, after which a sheet containing two tables was distributed among the participants. The first table included information on the students’ final grades in different subjects, separated by gender, based on information from the 2015–2016 academic year provided by the schools (see Supplementary Appendix Table 1). This table showed that, on average, girls performed better in all subjects, including those traditionally considered masculine, such as mathematics, technology, and physics. The second table contained data from the previously mentioned larger quantitative longitudinal study, showing the mean differences between boys and girls in their self-perception of ability based on a 7-point Likert scale (see Supplementary Appendix Table 2). This table showed that boys had a higher estimation than girls of their own abilities in subjects such as mathematics, physics, chemistry, and technology. Using these tables as the main basis of discussion, the focus groups were guided by open-ended questions designed to engage teachers and parents in discussion about the differences between male and female secondary students’ school achievement and self-perception of ability in different subject areas. The focus-group protocol included three broad categories of questions. In the first question, participants were asked to discuss their views on the data shown in the tables and how realistic they perceived this data to be. In the second question, participants were asked to list the subjects in which they thought each gender performed best. In the third question, participants were asked to give their opinion on the extent to which sexist beliefs about academic performance prevail in the classroom and in which subjects these beliefs are most prevalent. The same interview guide was used in all eight focus groups, all of which were audio-recorded and transcribed by a professional transcriptionist. The ethical study procedures were approved by the Institutional Review Board of the Universitat Oberta de Catalunya, and prior informed consent was obtained from the participants.

### Data Analysis

Data-driven thematic analysis on the interviews was carried out, following the approach described by Boyatzis (1998). Thematic analysis is especially suited for studies which address exploratory research questions, since the themes derive from statements made by participants rather than being defined previously by the research team. Three researchers (JS, MS, and SF) participated in the data analysis, which was conducted in three steps. First, all the interviews were read several times by JS, who identified and summarized the main themes. These themes were transformed into codes, and each code was assigned a label, a definition, and inclusion and exclusion criteria. The content and structure of the code book was subsequently discussed with MS and SF to check for consistency between the codes and the summarized themes. Any resulting disagreements between the three researchers were mediated through discussion and any necessary revisions were made. Second, the code book was imported into the software—NVivo 12 (QSR International, 2020)—used by JS to code the interview transcript. Text passages associated with the research questions were coded line by line by the researcher. Once the first stage of coding was completed, the codes were rigorously reviewed by MS and SF. Third, a reiterative approach was used to sort, collate, and combine the codes into main themes. The resulting themes were examined by MS and SF to confirm their relevance to the research questions. Finally, the “matrix coding query” function of NVivo was used to identify patterns in the coded data across the study participants. This last step allowed us to establish interpretive validity and within-case generalizability (Maxwell and Chmiel, 2014).

## Results

### Gender Differences in Academic Performance

Thirty-five parents (29 mothers and 6 fathers) referred to this issue, compared to 5 out of 10 male teachers, 13 out of 24 female teachers, and only 8 out of 21 participants with a STEM profile. In general, both parents and teachers were somewhat surprised at the fact that girls reported higher scores in all subject areas. They recognized that gender differences are higher in subject areas that require more dedicated study, such as biology, geology, and social sciences, or those that require verbal competences, such as Spanish, Catalan, and English.

Above all in more academic subject areas that require study. Biology and Geology. Social Sciences. There is a difference of over […] half a point, and look, of nearly one point. Biology and Geology, English, subjects associated with verbal skills. (Female school counselor, 52, Psychology graduate. FG3.)

However, both teachers and parents were particularly astonished to observe that girls have higher grades than their male counterparts in subjects traditionally associated with masculine roles, such as mathematics, technology, physical science, and chemistry.

It is surprising… the belief that boys are much better than girls at mathematics or scientific subjects is not true. (Female teacher, 42, school counselor, Psychology graduate, 10 years teaching experience. FG1.)

Generally speaking, they referred to several factors to explain that girls show higher scores in all subject areas. In both groups, participants agreed that girls mature earlier than their male counterparts during the compulsory secondary school years. This maturity enables girls to be more constant and structured; they are more responsible, disciplined, and focused; manage their time better; take more interest in academic endeavors; and are more realistic with the educational setting and the importance they give to academic outcomes. Boys, on the other hand, are more absent-minded.

They are more constant in their studies and demand a bit more from themselves. This can be associated with that maturity level. They reach that maturity level earlier. (Mother, 53, nurse, with one daughter. FG6.)

According to most teachers, these differences in school performance tended to diminish in post-compulsory secondary education (in Spain, Baccalaureate), when boys’ and girls’ performances even out.

I think that, during ESO [Spanish compulsory secondary education], this is clear, except for some boys who are very good and are able to perform well. But the girls perform better on average […]. I don’t know, I have the impression that, during ESO, boys do not take much interest whereas, in Baccalaureate, at least the brightest boys take more interest in what they do. (Female English teacher, 56 years old, 33 years of teaching experience. FG3.)

In some of the groups, a small number of participants discussed the idea that girls tend to study more and are more inclined to have more of a “culture of endeavor” than their male counterparts. However, for some participants, these gender differences in school performance were not attributable to boys’ lack of effort.

I believe there is a big difference in the way they work, but not a lack of effort in boys. (Mother, 46 years old, computer scientist, one son and one daughter. FG6.)

Other participants discussed biological and anatomical brain differences between men and women, which led to discussion of types of intelligence and the assertion that girls possess greater emotional intelligence than boys, while boys have greater problem-solving intelligence. According to the following testimony of a language female teacher, these different types of intelligence predisposed girls and boys to achieve different competences, even in areas like language, in which women are supposed to excel.

[…] girls’ emotional intelligence helps them to acquire a higher level of language than boys, in the aspect of the subject related to giving opinions and expressing emotions, or reasoning. Emotionally speaking, girls are more mature than boys. However, when it comes to my subject […] the gender gap gets smaller. Part of this subject requires that intelligence be focused on problem-solving. And the boys do better in these tasks, they are able to do them better. (English teacher, 55 years, with 34 years of teaching experience. FG2.)

Some participants in both groups questioned their role as part of the socialization process that promotes this division of roles outside school hours, whereby girls are encouraged girls to study, engage in responsible activities, and over-perform, while boys are encouraged to be more proactive, empowered, and oriented to competitiveness through activities like sports or playing video games.

[…] girls have been steered toward study, whereas boys are encouraged to do more things […]. For instance, they do more sport, play video games. (Mother, 47, customer care agent, with two daughters. FG8.)

### Gender Differences in Self-Perception of Ability

The participants raised several aspects associated with motivation to explain the observed gender differences in self-perception of ability. For instance, they commented that boys were more interested in subjects related to science and technology, or that they felt more competent than girls in these subject areas. Girls, on the other hand, seemed to feel more insecure, less competent, and comfortable and had less self-esteem than boys in these subject areas. As stated by one female teacher of Spanish, whereas boys grounded their self-confidence in their capacity, girls’ self-confidence was based on their endeavor (hours of study). This demonstrates that boys have a higher self-confidence in their intellectual capacity.

I believe that boys are more positive, have greater confidence in their intelligence. Among boys, there is feeling that there is no need to study; boys have confidence in their ability. Girls base their confidence on how much they study. (Teacher of Spanish, 56, 33 years of teaching experience. FG3.)

However, technology teachers recognized that girls were frequently attributed less competence by their male peers in the most technical activities developed in the classroom. According to these teachers, attitudes like these led girls to internalize that they were not capable enough to develop technological tasks in practical sessions like the ones in the workshop. The following ideas expressed by a female Technology teacher illustrate the existence of these prejudices about girls’ lack of technological competence.

It is true that when it comes to using the machines […], the girls, in general, remain withdrawn and think that they could not do it. Many times I have had to call the boys out for saying, “I don’t want to work with girls because they don’t know anything.” And I look at them and reply, “Okay, and what am I? I am a woman who teaches you technology.” And they reply, “Well not you, you’re the teacher.” There is still a lot of prejudice in technology, in the workshop, in relation to women supposedly having no technological competences and the worst of it is that girls just assume they do not have enough technological competences. (Female technology teacher, 47, Physical Science graduate, 22 years of teaching experience. FG4.)

For some participants, girls’ lower self-perception of ability in STEM was also associated with societal expectations and how these were higher for women than for men.

Expectations are greater for women because I believe that society influences us and we are told we have to work harder to achieve the same results. This is something that our mothers have probably unconsciously instilled in us. (Mother, 56, nurse with one daughter. FG4)

The promotion of different skills in boys and girls together with the disparity of social expectations becomes salient in subjects and activities associated with masculine gender roles, such as technology. In this context, sexist patterns of behavior are reproduced in the classroom. Boys tend to take the initiative, whereas girls self-exclude themselves from participating.

[…] Purely academically speaking, when pupils are working in a team, if I leave the tool kits on the table in a mixed group, the hammer and the saw, a girl never picks them up. That is, in order to have equality-based learning, I have to intervene […] Not because he knows more, but because the keyboard and the mouse have to be shared and the boy will take the initiative. Because we live in a society that fosters initiative in boys and reflection in girls. (Technology teacher, 48, Physical Science graduate, 16 years of teaching experience. FG2.)

Some mothers in a group of only mothers acknowledged that they sometimes unconsciously demand more from their daughters and are more tolerant with their sons. In their view, the gender socialization received during childhood influences the “not always conscious” reproduction of certain gender inequalities.

Well, we have experienced that with our own children, but we are trying to fight it. Although I try to treat my two children the same, I have realized that I sometimes don’t. Unconsciously, my own experiences, the way I was raised, make me more tolerant with my son than with my daughter and I demand more from my daughter. I sometimes find it hard. (Mother, 46, psychologist, with one son and one daughter. FG8.)

This disparity of gender socialization demands can also be observed in social media such as Facebook or Twitter, publicity, and the mass media, where messages congruent with gender roles and stereotypes are frequently addressed to boys and girls.

Last week I saw an article on Facebook about a study with primary children who observed posters advertising products targeting men and women, and looked at people’s perception of the men and women depicted in those posters. Women were depicted in terms like “sad,” “skinny,” “nobody loves her,” “she has been beaten,” and so on. And men were described in terms like “I want to be with him,” “Wow, so handsome,” “Look how confident they are” (Mother, 46, psychologist, with one son and one daughter. FG7.)

Some teachers questioned the role played in all this by schools and, by extension, themselves, given their ongoing beliefs regarding the prevalence of family education. One female teacher affirmed that, in transversal subjects like languages (where the incorporation of topics like racism or gender is frequent), the opinion of the family prevails.

The ideas that they have at home about racism, gender, and other topics are those that prevail. In most cases, not 100%, you do convey something to them, but what they experience in their family always prevails, in subject areas traditionally associated with the feminine gender role, like languages. (Female guidance counselor, 55, 27 years of teaching experience. FG4.)

Several participants discussed the weight of biological factors (instinct) versus those associated with “social DNA” to explain existing gender gaps in students’ perception of competence in different subject areas, and the implications that this could have when they conduct any concrete measure on aspects supposedly biologically marked.

There are two aspects, social and biological, right? So one can intervene and develop multiple tasks and activities and plan projects to attempt to minimize these differences. That is at a societal level, though I am not sure of the impact on society and how it will turn out. There is something in social DNA. But then, the biological issue, and for me the most important issue, is that equality rests also on understanding the difference […] I don’t know to what extent we should demonstrate the biological implications of all these. I can strive to ensure that someone picks up the hammer, a person who by instinct does not want to do it. Then, I could decide to intervene in a thousand aspects, but maybe I am also conditioning that person’s happiness. Maybe I will make that person unhappy, because that person (a girl or a boy) by instinct does not want to pick up the hammer […]. (Male Physical Science and Chemistry teacher, 38, Chemistry graduate, 11 years of teaching experience. FG2.)

At the same time, according to the views of a group of female teachers and a few parents, girls were more motivated to achieve good academic results, whereas boys settle for passing. This leads girls to make high demands of themselves and invest more effort in schoolwork than their male counterparts. In addition, because girls are perceived as hard workers when they achieve good grades, they too put their results down to hard work.

I used to get good grades, but I did not consider myself clever, I considered myself a hard worker. (Mother, 43, Cinema and Advertising graduate, with one daughter. FG7.)

Most of the teachers and parents agreed on the need for measures to change and improve girls’ self-perception of ability in STEM subject areas traditionally associated with masculine roles, such as technology or the “hard” sciences. A few teachers also believed that it is key to correcting boys’ overestimation of their competence in STEM domains.

I believe that what we have to work on is improving girls’ perception of their ability in math, physical science, and chemistry, and technology. Because […] this is a problem of perception, and perception can be corrected. Girls must be aware of this. (Father, 52, university teacher, with two sons. FG6.)

Across all groups, several solutions to be developed within the school context (starting from primary school) were discussed to resolve these gender disparities in students’ self-appraisal of ability. For instance, acting on the distribution of school spaces in the playground, not allowing boys to take ownership of these spaces to play football or engage in any activity that involves movement, relegating girls to a smaller space to talk or engage in activities with few movement opportunities.

I believe that school, of course during high school, but above all in primary school, should be the target of this work. From not letting boys occupy the playground playing football, leaving the girls with a corner, to the school, the classroom itself, so that when a female student performs well or does something that shows her knowledge on a topic, teachers acknowledge it publicly and empower her, and don’t tell her, “Return to your seat.” (Mother, 43, Cinema and Advertising graduate, with one daughter. FG7.)

Equally, the role of teachers in combating the reproduction of social roles in traditional areas like technology was also discussed. In this context, technology teachers acknowledged that, in the face of girls’ underestimation of their STEM competences, they can play an important role in setting up practical interventions to raise girls’ self-esteem and self-consideration of their STEM abilities.

There are very good people, who do not consider themselves good because of the influence of gender, and I think I can intervene there. This happens in Technology […]. I have to encourage girls, whereas I have to tell some boys that they are not quite as fantastic as they believe. (Male technology teacher, 49, Physical Science graduate, 24 years of teaching experience. FG2.)

In addition, a number of teachers questioned why schools are not offering a paradigm to neutralize the sexist, gender inequality messages that young people receive from different sources, such as the mass media. Accordingly, these societal messages reinforced the idea that what men do has a greater value than what women do, despite many women occupying jobs of responsibility in educational institutions.

It strikes me very much that schools do not play a more paradigmatic role, since we have increasingly been seeing many women present in education, more women holding posts of responsibility. In the previous managerial team, the director and the academic secretary were women. There are more female heads of department than males. Unfortunately, this has not had exemplary value because they continue to have sexist attitudes, even in the classroom, in the way they treat each other. Then the images they receive from society, the mass media and, I believe, other realities, is that men have a superior value to women […] Men continue to rule, men hold the highest positions in government, the Supreme Court, and this [referring to women holding positions of responsibility in education] does not permeate into society. (Male Spanish teacher, 45, 28 years of teaching experience. FG4.)

On the other hand, mothers and fathers deliberated about the important role that families play in counteracting the weight that society has in the transmission and reinforcement of gender inequalities.

As mothers we can transmit much to modify the stereotypes that are clearly shown in the surveys. We have to start working individually […] We have the theory, but then the collective unconscious, that social model, is more present than we wanted, which tells us we should never let our guard down and remain vigilant […] There is still much work to do to counteract it all. (Mother, 49, primary school teacher, with one daughter. FG7.)

### Inconsistency Between Actual Grades and Self-Perception of Ability

Only two out of 10 ten male teachers commented on this aspect. Similarly, only five out of 24 female teachers, ten out of 32 mothers, two out of seven fathers and five out of 21 participants in STEM tackled the issue. A number of fathers and mothers were especially astonished at with boys’ and girls’ predisposition to respectively underestimate and overestimate their abilities in traditional masculine subjects like technology, physical science, and chemistry, while their grades reflected the contrary.

To me, what is striking, and I like it, is that this happens in those subjects we think boys are better at than girls, such as mathematics. (Mother, 52, lawyer, with one son. FG7.)

To explain this discrepancy, mothers in most groups mentioned the prevalence of stereotypes and traditional gender roles. For instance, the perception that boys are more suited to sciences than girls was associated with the belief that boys are more competent in STEM than girls.

That is, it is viewed more favorably that boys enroll in scientific studies, and so we become convinced that boys have more of the qualities needed for science, when it is not a proven fact. (Mother, 57, Biology graduate, environment expert, with one daughter. FG7.)

Both parents and teachers agreed on the idea that raising girls’ self-perception of ability and self-esteem, in both academic and the family settings, would be essential to change these gendered predispositions. However, some teachers also remarked on the need to work on changing boys’ overestimation of their STEM abilities.

It is key that boys tend to perceive themselves as better, when their grades reflect the contrary. (Male Philosophy and Ethics teacher, 65, Philosophy graduate, 35 years of teaching experience. FG4.)

Parents and teachers granted that this discrepancy between grades and self-perception of competence provides an explanation for existing gender differences in decisions relating to courses and academic itineraries. That is, girls opt for biology and language-related subjects and itineraries, whereas boys choose science and technology-related subjects and itineraries.

Why do more boys choose technology than girls? I believe that the level of abstract and logic reasoning is more tied to gender and I don’t know how to explain whether it comes from early education. A high percentage of boys, when you launch the topic for a project, “We are going to do this,” they are the first to look for ideas, whereas the girls do nothing. (Female Technology teacher, 45, Engineering graduate, 16 years of teaching experience. FG2.)

In this regard, a number of teachers commented that parents’ opinions carry greater weight than the students’ own.

I am afraid that, if the family believes the girl is only worthy of being a hairdresser, you can tell them she can be an engineer she will not make it. (Male math teacher, 58, with 33 years of teaching experience. FG4.)

## Discussion

The present study contributes to the literature with a qualitative study of parents’ and teachers’ views of the persistence of gender stereotypes about boys’ and girls’ academic competences. For this reason, the engagement of the entire educational community, through both parents and teachers, in the fight against the prevalence of these gender stereotypes is crucial. These prejudices are harmful for everyone, but especially for boys and girls who do not fulfill societal expectations about their competences. The present research provides evidence of the fallacy regarding girls’ low degree of science and technological competences, as well as the lack of truth in the assumption that all boys have the same high level of technological competence. In addition, teachers’ and parents’ opinions about the incongruence between self-perception of ability and school achievement, above all in STEM subjects traditionally dominated by men, is another major contribution of the present study.

The study further suggests that it will be essential, in Spain at least, to involve parents (particularly mothers, since they seem to play a more active role in the educational dynamic) and secondary teachers in the design and development of future research and interventions aimed at preventing students from having biased ability self-concepts and promoting accurate ability self-concepts in both genders. However, empowering girls’ participation and perception of ability in STEM domains and activities is critical if we are to attract and retain more women in STEM fields. The same would be true for boys in occupations oriented toward care provision or the humanities.

### Gender Gaps in Academic Performance

The results of the present study demonstrate that many parents and teachers were not fully aware of the existence of gender differences in scholastic achievement and girls’ better academic performance. They were especially surprised by the evidence of girls’ higher performance in STEM subjects frequently associated with masculine roles.

They acknowledged that this gender gap occurred because girls work harder than boys, especially during junior secondary education, given that girls mature earlier. Some teachers also remarked that these gender differences in school performance tended to become smaller during senior secondary education. This line of argument reinforces postulates of the gender intensification hypothesis (Hill and Lynch, 1983). According to this hypothesis, following the commencement of puberty, changes in boys’ and girls’ appearance triggers increased social pressure from peers and adults to behave in traditional, gender-differentiated ways. This intensification disappears during mid- and late adolescence when adolescents are more flexible.

For some parents and teachers, these gender differences in school performance could also be associated with innate brain differences that may provide girls and boys with different intellectual abilities. Whereas girls have more emotional intellectual competences, boys have a problem-oriented intelligence. These traditional views of intelligence could be considered fixed entities in boys and girls by some of the participants (Dweck, 1999). This could have important implications for the way in which boys and girls conceptualize and calibrate their own talents, and the efforts they can make to develop and consolidate their different intellectual abilities.

Likewise, most parents and teachers discussed their role as part of the socialization process in the development of different gender roles associated with the importance attached by boys and girls to school achievement. However, there was no discussion of how they might contribute to changing the observed gender differences in school performance. This confirms other studies conducted in Spain (Sáinz et al., 2012), suggesting that teachers and parents should receive training with a gender perspective.

### Gender Differences in Self-Perception of Ability

In light of the gender differences in students’ self-perception of ability, many of the participants employed different personal and motivational arguments, such as interest, self-confidence, comfort, and self-esteem in relation to the different subject areas (Eccles, 2007, 2009). Remarkably, the debate was very much focused on the gap in self-perception of ability in STEM subjects that favor boys, such as technology, but not in those that favor girls, such as languages, or biology and geology. Interestingly, a female teacher clearly explained how girls base their self-perception of ability and self-confidence on the effort they make, whereas boys do so based on their self-perceived capacity. This line of argument is congruent with research which draws on attribution theory and postulates that, whereas boys’ achievement is more often attributed to capacity, girls’ achievement is generally attributed to effort (Eccles, 1987, 2007). Mainly female teachers also discussed how, whereas girls in general learn to focus on schoolwork, boys convince themselves that passing is enough. This suggests that school should design interventions oriented toward addressing the difference in the value attached by adolescent girls and boys to school achievement.

Technology teachers, however, went further and recognized that girls’ lower perception of ability in technology is associated with current prejudice about girls’ technological competences (Sáinz et al., 2012, 2019; Shin et al., 2016). For these teachers, girls internalize this low expectation of their ability in STEM, turning it into a self-fulfilled prophecy (Jussim et al., 1996; Rosenthal and Jacobson, 2003). This view corroborates research on stereotypes about women’s supposed lack of technical skills (Sáinz et al., 2019) and how the socialization of gender roles shapes boys’ and girls’ self-perceptions of ability (Eccles, 2007; Simpkins et al., 2012). In addition, according to many participants, girls’ low self-perception of ability in STEM subjects was associated with the high expectations demanded by society of women in general. In other words, women have to try harder if they want to attain the same goals as their male counterparts, and this is applicable to all facets of our lives.

Participants also discussed the importance of gender socialization and how the dissimilar messages provided by different agents can be aligned with gender stereotypes that reinforce gender differences in self-perceptions (Eccles, 1987, 2007). Interviewing parents with children from both genders reflected on the way they behave differently with their children according to their gender. In this regard, some mothers recognized that they often unconsciously established unequal demands for daughters and sons. This confirms the findings of a recent scoping review which provided qualitative evidence of the particular importance of mothers in teaching and enforcing stereotypical gender roles, especially with their daughters (Kågesten et al., 2016).

In addition, the messages young people receive from the mass media (including social media) about different ways of depicting masculinity and femininity were also discussed. Whereas some of the participants questioned their role as parents or teachers in fighting against the transmission of stereotypical portrayals about academic competence, others seemed to consider that “other people” (not themselves) played a more important role than themselves in this (Sáinz et al., 2012).

A number of participants also discussed the interplay between biological and social factors in shaping gender differences in self-perception of STEM ability. In this respect, they discussed different ways to reduce these gender differences. All agreed on the importance of working on increasing girls’ self-perception of ability in STEM (Sáinz and Upadyaya, 2016; Sheldrake, 2016), while some teachers also pointed out the need to modify boys’ tendency to brag about their STEM abilities (Guimond and Roussel, 2001).

Also worthy of attention is the way in which some teachers questioned the role played by schools and, by extension, themselves, in combating the gender roles and stereotypes present in current society. It is particularly interesting that they flagged up the fact that education (normally associated with feminine gender roles) lacks in social value compared to other sectors (like politics or economy), given that society attaches greater value to what men do as opposed to women. This might also explain why none of the focus groups discussed the implications of a deficit of boys in non-STEM fields such as the humanities, and how to encourage the participation of boys in professions oriented toward health and care provision, where the presence of women predominates.

It is interesting that a number of teachers and parents (especially those coming from technological fields) provided insightful guidelines for intervention that have also been identified in the literature, such as, for instance, preventing boys from taking control of school spaces such as the playground (Clark and Paechter, 2007; Lyttleton-Smith, 2019), or deterring boys and girls from assuming traditional roles within the classroom—for instance, in practical workshops in technological fields (Sáinz et al., 2012). Likewise, some groups of parents also discussed their role in changing boys’ and girls’ predisposition to respectively over- or undervalue their STEM abilities (Guimond and Roussel, 2001; Sáinz and Upadyaya, 2016). Unlike the teachers, however, they did not discuss concrete actions to employ at home to change these predispositions, though some did reflect on the need to modify societal stereotypes in the home.

### Incongruence Between Self-Perceptions and Performance

The parents seemed to be more astonished than the teachers by the incongruence between self-perceptions and performance. They were particularly shocked by the predisposition of boys and girls to respectively over- or underestimate their competences in STEM subjects.

The prevalence of gender stereotypes about men’s and women’s abilities and traditional roles was a common topic for discussion in all the focus groups. However, though participants from the different groups questioned their own role in shaping accurate perceptions of boys’ and girls’ academic competences, teachers insisted on the importance of the role of families in shaping young people’s self-perceptions of ability and academic decisions such as study choices (Sáinz and Upadyaya, 2016). Most of these decisions were taken in the course of secondary schooling, where social pressures to conform to gender roles are very salient (Eccles, 1987, 2007).

Most parents and teachers associated young people’s inaccuracy in the evaluation of their academic competences (with a particular focus in STEM fields) with existing vocational segregation. That is, whereas girls are highly represented in STEM studies related to the provision of health, they remain underrepresented in technological STEM programs and physical science (Spanish Ministry of Education [MEFP], 2020). In this regard, teachers affirmed that parents’ opinions have greater influence over young people’s study choices.

The fact that most of the discussion among parents and teachers was focused on STEM fields also informs us about the perceived importance of STEM fields in current society. In Spain, STEM professions tend to be associated with much greater prestige in terms of salary, academic difficulty and social consideration than other professions (Sáinz et al., 2016).

### Future Directions

With regard to teachers, it is thus essential to identify the major shortcomings in primary and secondary teacher training and encourage the incorporation of gender as a prerequisite in their curricula in order to achieve inclusive, plural, and diverse models for teaching practices. Teacher training programs should analyze the implications of gender inequalities in students’ self-perceptions of ability in the official school curriculum, materials, and teacher practices.

The opinion and experiences of technology teachers can inspire the design of measures to neutralize gender differences in self-perception of ability in particular and in school dynamics in general. These experiences can also inform about concrete teacher practices that could be implemented to empower girls’ competences not only in STEM but also in other non-STEM subject areas, such as economics. In addition, these teacher practices could also be worth sharing in order to counteract boys’ tendency to brag about their STEM abilities. Future research should analyze the long-term effects and implications of these inaccuracies in boys’ and girls’ career pathways, from primary school through to the transition to work.

Further research involving parents is also required. Since one of the challenges for researchers studying parental socialization seems to rest on the separation of parent–child influences from parent–child influences (Leaper, 2014), studies examining both influences are essential. More interventions involving parents and teachers to promote realistic views of their competences in boys and girls are also required.

In addition, given that research on gender differences in different facets of intellectual achievement has provided the justification for policy decisions such as funding for sex-segregated education (Lindberg et al., 2010; Voyer and Voyer, 2014), future research is still required to define the main factors associated with gender differences in school performance and self-perceptions of ability.

### Limitations

Limitations of the present study may be attributed to the lack of use of a non-focused interview guideline. However, the exploratory nature of this research required that parents and secondary teachers expressed their views in an open way. Another limitation of the present research is the underrepresentation of fathers. Future research should incorporate more men and their perspectives on these issues. Engaging more men in these issues is highly requested.

## Data Availability Statement

The raw data supporting the conclusions of this article will be made available by the authors, without undue reservation.

## Ethics Statement

The studies involving human participants were reviewed and approved by Institutional Review Board of the Universitat Oberta de Catalunya. The ethics committee waived the requirement of written informed consent for participation.

## Author Contributions

MS conceptualized, designed, and coordinated the realization of the study, wrote the first draft of the manuscript. SF wrote most of the method section and made insightful comments with regard to the justification of the study, whereas JS contributed to the development of the results section. All the authors contributed to manuscript revision, read, and approved the submitted version.

## Conflict of Interest

The authors declare that the research was conducted in the absence of any commercial or financial relationships that could be construed as a potential conflict of interest.
