# Supplementary material for: Parent and Teacher Depictions of Gender Gaps in Secondary Student Appraisals of Their Academic Competences
Source: Front Psychol. 2020 Sep 25;11:573752. doi: 10.3389/fpsyg.2020.573752 (PMC7545902; doi:10.3389/fpsyg.2020.573752)
Supplement: Supplementary file 1 [file Table_1.DOCX]

Table 1. Students’ grades in different subjects by gender (Academic course 2015-2016)

| **Subject** | **Boys** | **Girls** | **Difference** |
| --- | --- | --- | --- |
| Mathematics | 5.01 | 5.42 | -.41 |
| Spanish | 5,35 | 5.98 | .-63 |
| Catalan | 4.91 | 5.66 | -.75 |
| English | 5.58 | 6.38 | -.8 |
| Physics and Chemistry | 5.41 | 5.98 | -.57 |
| Biology and Geology | 5.77 | 6.73 | -.96 |
| Social Sciences | 5.93 | 6.43 | -.5 |
| Technology | 5.85 | 6.33 | -.48 |

Grades range from 0 to 10

Table 2. Self-perception of ability by gender

| **Subject** | **Boys** | **Girls** | **Difference** |
| --- | --- | --- | --- |
| Mathematics | 4.42 | 4.20 | +.22 |
| Spanish | 4.33 | 4.85 | -.52 |
| Catalan | 4.01 | 4.83 | -.81 |
| English | 4.92 | 5.22 | -.30 |
| Physics and Chemistry | 4.07 | 3.80 | +.27 |
| Biology and Geology | 4.34 | 4.75 | -.39 |
| Social Sciences | 4.69 | 4.75 | -.06 |
| Technology | 4.79 | 4.08 | +.71 |

Values range from 0 to 7
